# Exploring the prevalence of chronic diseases and health status among international Hajj pilgrims

**DOI:** 10.1371/journal.pone.0317555

**Published:** 2025-04-01

**Authors:** Osama Samarkandi, Fahad Alamri, Ghadah Alsaleh, Lamees Al Abdullatif, Jumanah Alhazmi, Mohammed Basnawi, Waleed Alazmy, Anas Khan

**Affiliations:** 1 Department of Basic Science, Prince Sultan College for Medical Emergencies, King Saud University, Riyadh, Saudi Arabia; 2 Global Center for Mass Gathering Medicine (GCMGM), Ministry of Health, Riyadh, Saudi Arabia; 3 Department of Medicine, College of Medicine, Umm Al-Qura University, Mecca, Saudi Arabia; 4 Department of Emergency Medicine, College of Medicine, King Saud University, Riyadh, Saudi Arabia; Mashhad University of Medical Sciences, IRAN, ISLAMIC REPUBLIC OF

## Abstract

**Background and aims:**

Pilgrims with pre-existing Chronic Diseases are at high risk of physical stress that can lead to unexpected health outcomes, including multiple visits to the hospital, organ failure, or even death. In addition, the risk of mortality related to Chronic Diseases increases during mass gatherings due to these same reasons. Therefore, this study aims to evaluate the Prevalence of Chronic Diseases and clinical symptoms among pilgrims during Hajj 2024 in Saudi Arabia.

**Methods:**

During the 2024 Hajj rituals, cross-sectional questionnaires were administered to pilgrims at the Jeddah International Airport. The questionnaire contained questions on sociodemographic information, the presence of Chronic Diseases, types of Chronic Diseases, and clinical symptoms.

**Results:**

A total of 1920 pilgrims were included in the study, with a mean age of 52.03 ± 13.43 and a median of 53 years. Among those, 49.4% were males. The prevalence of Chronic Diseases (CDs) was 36.3% (n = 697). Among those, 16% (n = 308) of them reported hypertension, followed by diabetes 11.6% (n = 222), asthma (4%, n = 76), and cardiovascular diseases (3.4% n = 65). The most common symptoms associated with Chronic Diseases were cough (36.5%, n = 700), followed by fever (21.8%, n = 419), and sore throat (21.2%, n = 407). The presence of diabetes was significantly associated with gender (p = 0.019), while hypertension was more common among male pilgrims (n = 829) compared to female pilgrims (n = 783) (p = 001). Furthermore, the presence of coughing was significantly associated with the age and educational level of the pilgrims (p = 0.001). On the other hand, headaches were more common among females compared to male pilgrims (p = 0.001).

**Conclusion:**

A considerable number of pilgrims suffer from Chronic Diseases, with hypertension being most common. Further interventions, such as education and management before Hajj, are recommended.

## Introduction

The Centers for Disease Control and Prevention (CDC) defines Chronic Diseases as ailments that persist for a year or more and require continuing medical attention, impede daily activities, or both [1]. The morbidity and mortality among Hajj pilgrims are crucial to health authorities to implement standard and immediate health facilities for pilgrims at holy cities [2–4]. Recent literature revealed that the number of pilgrims with Chronic Diseases performing Hajj could exceed 300,000 per year [2]. Individuals with pre-existing Chronic Diseases are at high risk of physical stress which can lead to unexpected health outcomes, including multiple visits to the hospital, organ failure, or even death [5]. For example, earlier literature revealed that Cardiovascular diseases and diabetes are the most common causes of death during Hajj [2] myocardial infarction, asthma, hypertension, and hyperglycemia were other causes reported among pilgrims by Alrufaidi et al [6].

The majority of deaths globally are related to non-communicable diseases which also contribute to mortality during the Hajj [2,4,7]. For example, a previous study revealed that 64% of pilgrims with cardiovascular diseases were admitted to the ICU and 66% of deaths related to cardiovascular diseases were reported during Hajj in 2008 [2,7,8]. On the other hand, another study revealed that mortality during Hajj among pilgrims was significantly associated with diabetes, hypertension, and cardiovascular diseases [2]. In addition, in 2021, Yezli et al stated in a systematic review that the pooled prevalence of hypertension and diabetes were 12.2% and 5.0% respectively. The prevalence of chronic respiratory, kidney, or liver disease, cardiovascular disease, cancer, and immune deficiency was generally low among pilgrims [9].

Hajj is a gathering of Muslims from 180 countries, and previous studies show that pilgrims with poor health status are at greater risk of developing infectious diseases [2]. Furthermore, the spread of the coronavirus, Middle Eastern respiratory viruses, influenza, and other similar contagious diseases may worsen the health of the pilgrims with CDs. To overcome the spread of various viruses and diseases, the Saudi government has implemented healthcare measures before coming to the holy cities, including vaccinations and maintaining the availability of health services for all pilgrims during Hajj [10–12]. A recent report published by a Saudi press agency in May 2024 revealed that the Madinah Health Cluster has prepared 18 hospitals and medical centers with over 20,000 healthcare staff to work in medical centers, along with advanced medical equipment, laboratories, blood banks, necessary Hajj vaccinations, and emergency care facilities to serve pilgrims during the Hajj season in 2024 [13]. This was done in conjunction with the Lancet Infectious Diseases in the Saudi Ministry of Health (MOH) which established mass gathering medicine techniques and prevention methods [14].

Hajj is a life-changing spiritual experience, and one of the pillars of Islam, individuals are obligated to perform at least Hajj at least once in their lifetime. It is estimated that around 2 million people around the world come to the two holy cities to complete the Hajj rituals. This crowded environment presents unique challenges to people suffering from Chronic Diseases in terms of rigorous physical activity which exposes the pilgrim to fatigue and exhaustion, especially if they are not regularly physically active. Therefore, the physical fitness of the body should be restored before Hajj. Not only that but due to the nature of the lunar cycle, Hajj can often occur in changing seasons from year to year. This can lead to environments where the seasonal movement has implications for the spread of disease and other health risks, challenging public health policy planners further. The congestion of people means that emerging infectious diseases have the potential to quickly turn into epidemics. Therefore, a pre-travel consultation with a healthcare professional is recommended to assess fitness for travel, with an emphasis on guidance, education, and risk stratification for all prospective pilgrims with a chronic condition. Healthcare providers and patients must be aware of the risks that could happen during Hajj and the importance of well-being to ensure pilgrims perform Hajj safely. This study was conducted to address the health consequences among diseased pilgrims and to design advanced health facilities for pilgrims with CDs. Therefore, this study aimed to evaluate the Prevalence of Chronic Diseases and clinical symptoms among pilgrims in the 2024 Hajj in Saudi Arabia.

## Methodology

A cross-sectional study was conducted among pilgrims during the Hajj season in 2024, using pre-validated structured questionnaires. Hajj pilgrims over the age of 18 years, being able to respond to the questionnaires, were included. Female participants, who were pregnant or in the post-partum period were excluded. Before data collection, the study protocol was reviewed and approved by the institutional review board at King Fahad medical city, global center for mass gathering medicine, Ministry of Health, Riyadh, Saudi Arabia (Reference Number- 24-289E). Furthermore, written informed consent was obtained before data collection. Respondents were informed that the data would be used solely for research purposes and that confidentiality and anonymity would be maintained throughout the research. In addition, respondents were given full rights to withdraw from the study at any point in the time. All the study protocols were performed according to the declaration of Helsinki guidelines.

### Sample size estimation

Similar to earlier studies [15–17] the sample size was calculated using the Raosoft® sample size calculator, with 95% confidence intervals (CI), a response distribution of 50%, and a 5% margin of error (ME) the targeted sample size would be 385 participants. Adjusting for the projected 10% attrition, the estimated final sample size is at least 424 participants. Although to improve the study’s statistical power and allow for more accurate estimates and detection of significant differences and to minimize sampling bias and error, seeking cooperation from participants in the pilgrimage to take part in the research, we were able to collect a total of 1920 fully completed questionnaires. Therefore, we included all 1920 questionnaires in the final analysis.

### Study tool and data collection

The questionnaires used for this study was adopted from earlier studies [2,12,18,19]. The questionnaires consisted of several sections. Section one included sociodemographic characteristics of the pilgrims such as age, gender, educational classification, and nationality. Section two collected information from the pilgrims about the most common Chronic Diseases, like diabetes, hypertension, obesity, cardiovascular diseases, chronic lung disease, asthma, and Chronic Diseases related to the central nervous system. The health status of the pilgrims was determined by asking presence of Chronic Diseases (Yes or No). The third section of the study collected symptoms associated with Chronic Diseases among pilgrims and further divided them into the most common symptoms, symptoms related to cardiovascular and gastrointestinal diseases, pain-related symptoms, and symptoms related to skin, eyes, and ears.

After the initial draft of the questionnaires, it was evaluated by the primary investigator and a researcher, later questionnaires in English language were subjected to pilot testing among randomly selected pilgrims (n = 20) to check the flow of the questionnaires and their content. Later the validity of the questionnaires was determined using Cronbach alpha, which was found to be 0.76, suggesting that questionnaires are valid and reliable to carry out the study. A data collection team was formed, consisting of trained researcher assistants (n = 6) who speak Arabic, English, Urdu, French, Bengali, and Hindi. Furthermore, if neither the data collector nor the pilgrims understood the language, there was a translator to assist in understanding the language from their accompanying Hajj campaigns.

Potential participants were approached randomly by a research team member from Jeddah at the departure lounges at International Airports in both cities. They were asked to participate in a study concerning the presence of Chronic Diseases and symptoms. Those who agreed were administered the English questionnaire by one of the trained research assistants who recorded the responses in a Google form. Data collection was carried out using a structured questionnaire administered by trained research assistants.

### Statistical analysis

**Fig 1:**
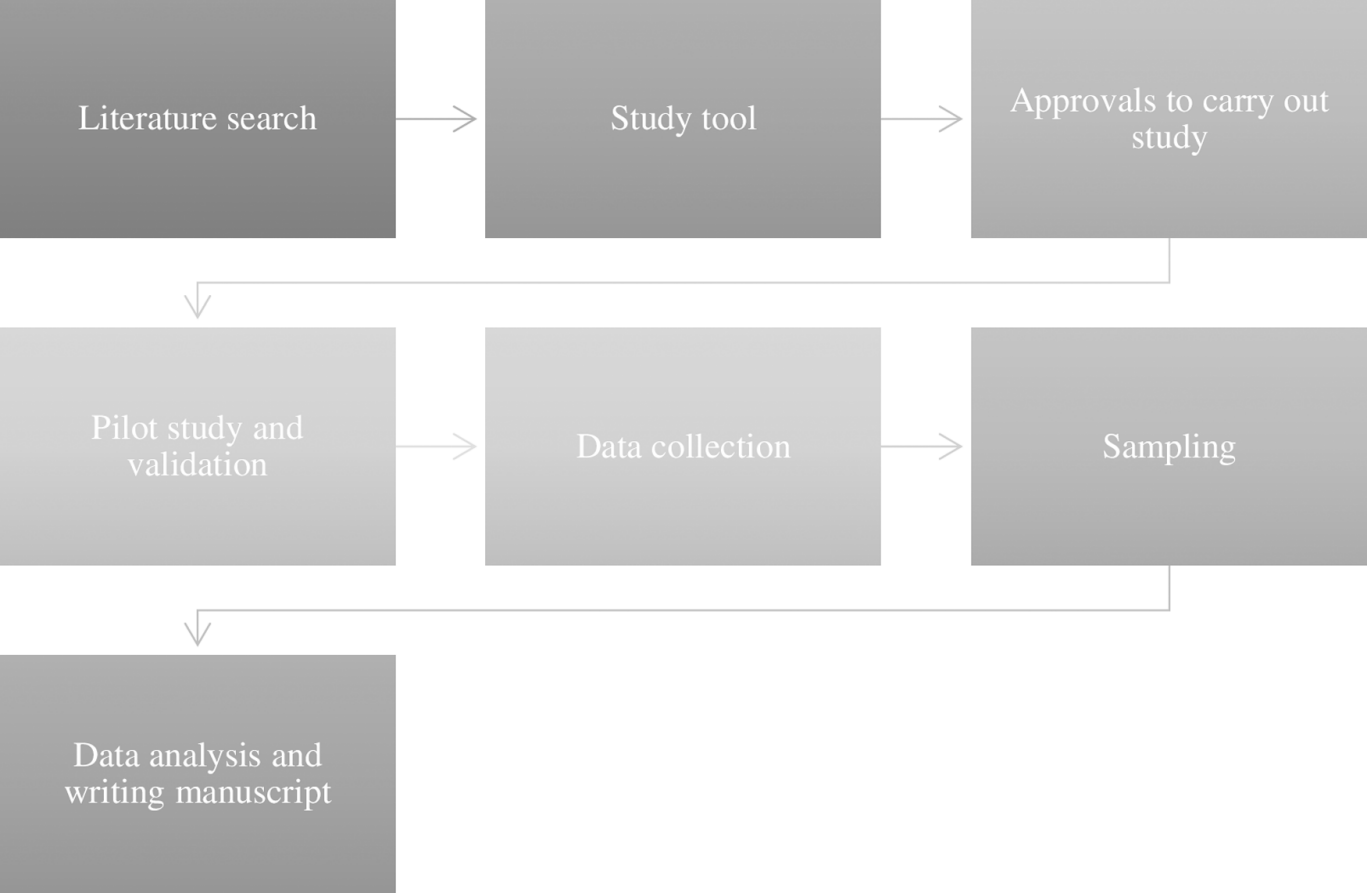
Flow diagram of the study.

SPSS statistical software will be used for data management and analysis. Descriptive statistics such as frequency (n) and percentages (%) were computed for all quantitative variables whereas for continuous variables the data was presented as mean and standard deviation. Categorical variables were compared using the Chi-square test or Fischer’s exact test as appropriate a p-value < 0.05 was considered as statistically significant. The detailed steps involved in the study design are depicted in Fig 1.

## Results

### Demographic characteristics of the pilgrims

**Fig 2:**
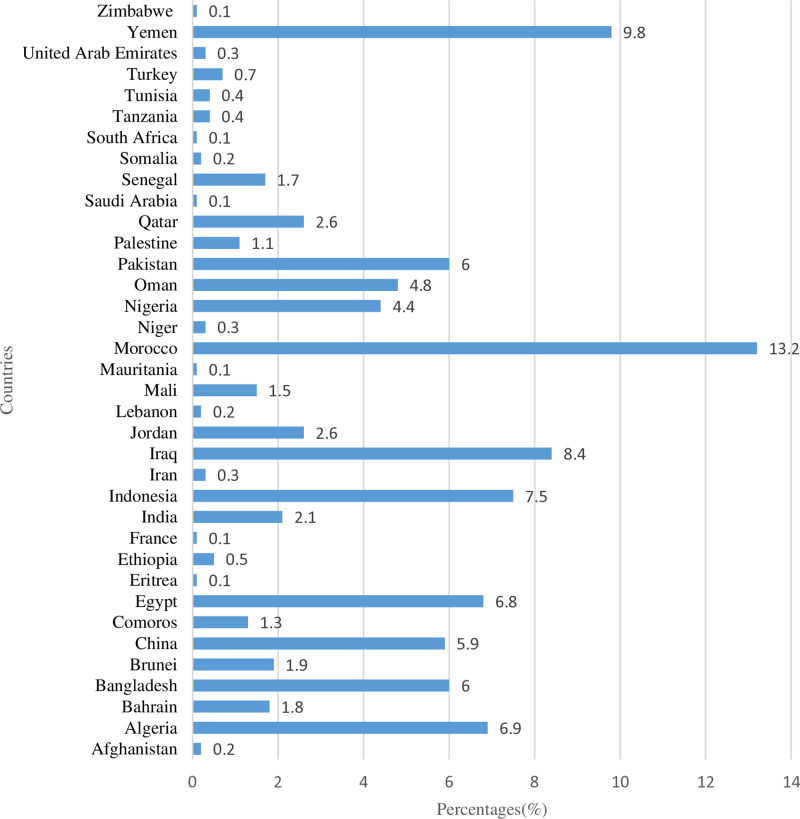
Geographic distribution of pilgrims.

A total of 1920 pilgrims were included in the study. Table 1 briefly summarizes the demographic features. The mean age of the pilgrims was 52.03 ± 13.43, median age was 53 years. The demographic data showed a gender distribution that was balanced, and the distribution of educational levels showed that 23.9% of participants (n =  458) had a university degree, with a substantial proportion of participants 36.3% (n = 697) reporting Chronic Diseases. The geographic distribution of the pilgrims showed a wide representation from various countries, with 13.2% frequency from Morocco, followed by Yemen at 9.8%, Iraq at 8.4%, Indonesia at 7.5%, and Egypt at 6.8% as shown in Fig 2.

**Table 1:** Demographic characteristics of the pilgrims (N = 1920).

Characteristics	Frequency (n)	Percentage (%)
**Gender**		
Male	948	49.4
Female	972	50.6
Age (Mean ± SD)	52.03 ± 13.43, Median 53 Range (0–80)	
**Educational level** ^*^		
Illiterate	145	7.6
Intermediate or Secondary	168	8.8
Able to read and write	341	17.8
University	458	23.9
**Do you have chronic diseases?**		
Yes	697	36.3
No	1223	63.7

* Missing response.

### Prevalence of chronic diseases

The current findings revealed that approximately 37% of the pilgrims reported Chronic Diseases. Among those, 16% (n = 308) reported hypertension, followed by diabetes at 11.6% (n = 222). A minor percentage of the pilgrims reported conditions such as asthma (4%, n = 76), cardiovascular diseases (3.4%, n = 65), arthritis 3.3%(n = 63), obesity 0.7%(n = 14), and renal diseases (0.3%, n = 6). The presence of Chronic Diseases among pilgrims is given in Table 2.

**Table 2:** Chronic diseases among pilgrims.

Characteristics	Frequency (n)	Percentages (%)
Diabetes		
Yes	222	11.6
No	1698	88.4
Diabetes on insulin		
Yes	65	3.4
No	1855	96.6
Hypertension		
Yes	308	16
No	1612	84
CVD		
Yes	65	3.4
No	1855	96.6
Liver disease		
Yes	11	0.6
No	1909	99.4
Asthma		
Yes	76	4.0
No	1844	96
Chronic lung disease		
Yes	7	0.4
No	1913	99.6
Epilepsy		
Yes	6	0.3
No	1914	99.7
Renal disease		
Yes	6	0.3
No	1914	99.7
Arthritis		
Yes	63	3.3
No	1857	96.7
Obesity		
Yes	14	0.7
No	1906	99.3

### Chronic diseases related to the nervous system

**Fig 3:**
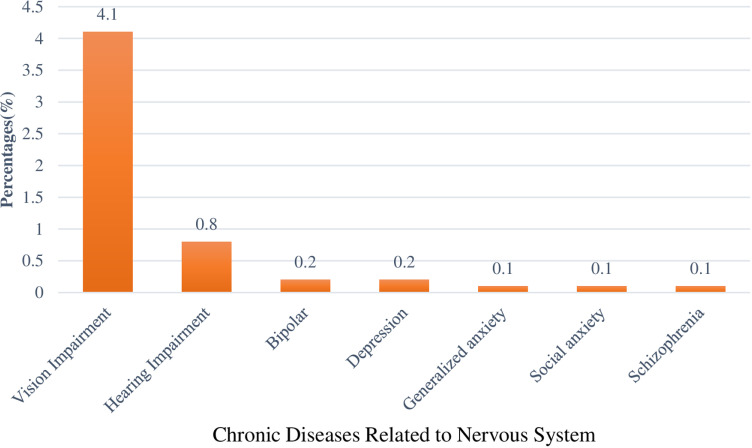
Chronic diseases among pilgrims related to the Central Nervous System.

In this study, 4.1% (n = 79) of the pilgrims reported having vision impairment, while 0.8%(n = 15) of them revealed hearing impairment. In addition, mental health diseases like bipolar disorder, depression, and various forms of anxiety were reported by less than 1% as shown in (Fig 3).

### Symptoms associated with chronic diseases

**Fig 4:**
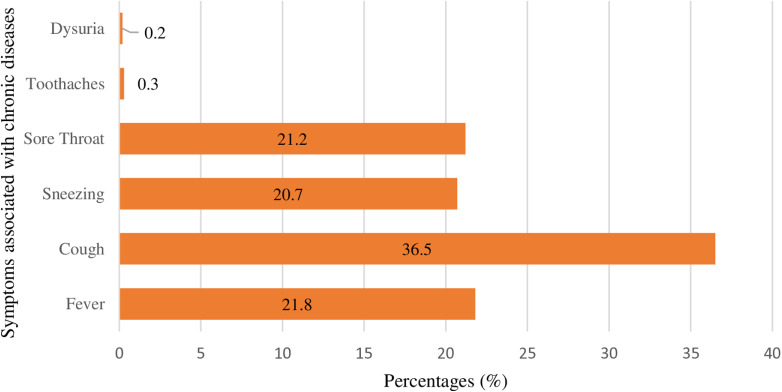
Frequency of distribution of most common symptoms associated with chronic diseases.

With regards to the distribution of the most common symptoms associated with Chronic Diseases included cough (36.5%, n = 700), followed by fever (21.8%, n = 419), sore throat (21.2%, n = 407), and sneezing 20.7% (n = 397) as shown in Fig 4.

### Symptoms related to cardiovascular and gastrointestinal diseases

**Fig 5:**
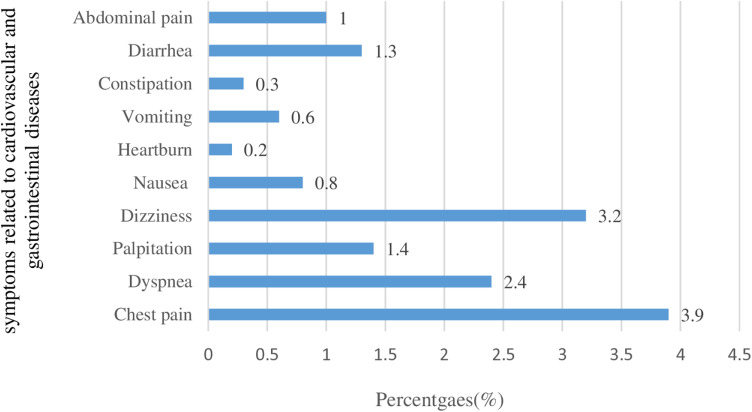
Frequency of distribution of symptoms related to Cardiovascular and Gastrointestinal Diseases.

Chest pain was most common among pilgrims 3.9% (n = 74), followed by dizziness 3.2% (n = 61), dyspnea 2.4% (n = 46), and palpitations 1.4% (n = 26). While diarrhea 1.3% (n = 24), abdominal pain (1%, n = 20), and nausea 0.8% (n = 16) as shown in Fig 5.

### Pain-related symptoms

The pain-related health symptoms experienced during Hajj were widespread, with 8.3% (n = 159) reporting foot pain, followed by headache or head pain 5.5% (n = 106), malaise and fatigue 4.7% (n = 91), body aches 3.1% (n = 59), and 2.8% (n = 54) of the pilgrims reported back pain (Table 3).

**Table 3:** Symptoms related to pain.

Characteristics	Frequency n	Percentage (%)
Headache		
Yes	106	5.5
No	1814	94.5
Malaise and fatigue		
Yes	91	4.7
No	1829	95.3
Back pain		
Yes	54	2.8
No	1866	97.2
Foot pain		
Yes	159	8.3
No	1761	91.7
Body aches		
Yes	59	3.1
No	1861	96.9
Muscles cramps		
Yes	13	0.7
No	1907	99.3

### Symptoms related to skin, eyes, and ears

**Fig 6:**
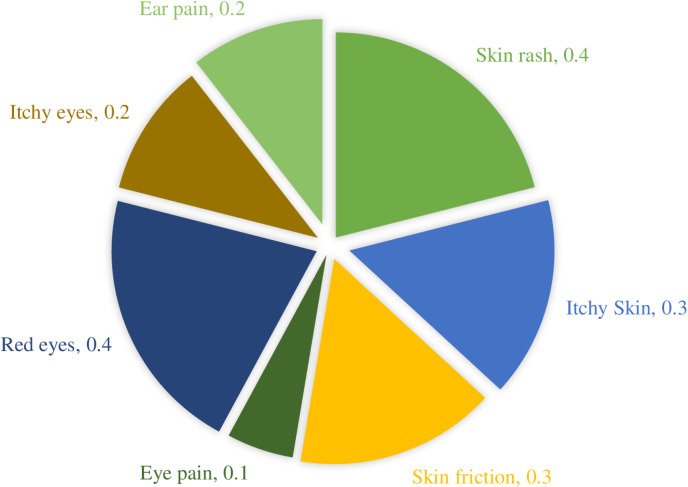
Symptoms related to skin, eye, and ear.

The current findings show symptoms related to skin, eyes, and ears were less frequent among pilgrims. Skin rashes were reported among 0.4% of them, followed by itchy skin at 0.3%, itchy eyes at 0.2% (n = 3), and ear aches at 0.2% (n = 4) as shown in Fig 6.

The findings revealed that hypertension and diabetes were significantly associated with gender, (p = 0.001) age group, (p = 0.001), and educational status (p = 0.001) of the pilgrims as shown in Table 4. However, the presence of CVD was not significantly associated with gender (p = 0.597), age (p = 0.001), and education (p = 0.005) was found to be significant with CVD (Table 4).

**Table 4:** Association between Chronic Diseases and pilgrim’s demographic characteristics.

Variables	Hypertension		p-value	Diabetes		p-value	CVD		p-value
	Yes n(%)	No n(%)		Yes n(%)	No n(%)		Yes n(%)	No n(%)	
Gender									
Male	119 (38.6%)	829 (51.4%)	0.001	93 (41.9%)	855 (50.4%)	0.018	30 (46.2%)	918 (49.5%)	0.597
Female	189 (61.4%)	783 (48.6%)		129 (58.1%)	843 (49.6%)		35 (53.8%)	937 (50.5%)	
Age									
22–30 years	2 (0.6%)	107 (6.6%)	0.001	1 (0.5%)	108 (6.4%)	0.001	1 (1.5%)	108 (5.8%)	0.001
31–41	15 (4.9%)	331 (20.5%)		18 (8.1%)	328 (19.3%)		3 (4.6%)	343 (18.5%)	
42–52	63 (20.5%)	438 (27.2%)		44 (19.8%)	457 (26.9%)		12 (18.5%)	489 (26.4%)	
53–63	125 (40.6%)	450 (27.9%)		91 (41.0%)	484 (28.5%)		20 (30.8%)	555 (29.9%)	
>64 years	103 (33.4%)	286 (17.7%)		68 (30.6%)	321 (18.9%)		29 (44.6%)	360 (19.4%)	
Education									
Missing	174 (56.5%)	634 (39.3%)	0.001	120 (54.1%)	688 (40.5%)	0.001	29 (44.6%)	779 (42.0%)	0.005
Illiterate	23 (7.5%)	122 (7.6%)		1697.2%)	129 (7.6%)		7 (10.8%)	138 (7.4%)	
Secondary	22 (7.1%)	146 (9.1%)		23 (10.4%)	145 (8.5%)		6 (9.2%)	162 (8.7%)	
Read & Write	39 (12.7%)	302 (18.7%)		36 (16.2%)	305 (18.0%)		19 (29.2%)	322 (17.4%)	
University	50 (16.2%)	408 (25.3%)		27 (12.2%)	431 (25.4%)		4 (6.2%)	454 (24.5%)	

Regarding symptoms associated with Chronic Diseases, the findings show that fever was more common among female (58.9%) pilgrims compared to male (41.1%), with it being higher among pilgrims aged between 53–63 years compared to other age groups. suggesting a significant association between fever and pilgrim’s characteristics as shown in Table 5. The presence of cough was significantly associated with the age and educational level of the pilgrims (p = 0.001). On the other hand, headache was more common among females compared to male pilgrims (p = 0.001). The detailed association between common symptoms associated with Chronic Diseases and pilgrim’s characteristics (Table 5).

**Table 5:** Association between Common symptoms associated with Chronic Diseases and Pilgrim’s characteristics.

Variables	Fever		p-value	cough		p-value	Sore throat		p-value	Headache		p-value
	Yes n(%)	No n(%)		Yes n(%)	No n(%)		Yes n(%)	No n(%)		Yes n(%)	No n(%)	
Gender												
Male	172 (41.1%)	776 (51.7%)	0.001	345 (49.3%)	603 (49.4%)	0.953	185 (45.5%)	763 (50.4%)	0.075	34 (32.1%)	914 (50.4%)	0.001
Female	247 (58.9%)	725 (48.3%)		355 (50.7%)	617 (50.6%)		222 (54.5%)	750 (49.6%)		72 (67.9%)	900 (49.6%)	
Age												
22–30	21 (5.0%)	88 (5.9%)	0.029	34 (4.9%)	75 (6.1%)	0.057	20 (4.9%)	89 (5.9%)	0.140	5 (4.7%)	104 (5.7%)	0.136
31–41	78 (18.6%)	268 (17.9%)		107 (15.3%)	239 (19.6%)		71 (17.4%)	275 (18.2%)		13 (12.3%)	333 (18.4%)	
42–52	109 (26.0%)	392 (26.1%)		183 (26.1%)	318 (26.1%)		113 (27.8%)	388 (25.6%)		24 (22.6%)	477 (26.3%)	
53–63	146 (34.8%)	429 (28.6%)		230 (32.9%)	345 (28.3%)		136 (33.4%)	439 (29.0%)		43 (40.6%)	532 (29.3%)	
>64 years	65 (15.5%)	324 (21.6%)		146 (20.9%)	243 (19.9%)		67 (16.5%)	322 (21.3%)		21 (19.8%)	368 (20.3%)	
Education												
Missing	197 (47%)	611 (40.7%)	0.033	343 (49%)	465 (38.1%)	0.001	170 (41.8%)	638 (42.2%)	0.003	44 (41.5%)	764 (42.1%)	0.774
Illiterate	24 (5.7%)	121 (8.1%)		33 (4.7%)	112 (9.2%)		19 (4.7%)	126 (8.3%)		5 (4.7%)	140 (7.7%)	
Secondary	25 (6%)	143 (9.5%)		43 (6.1%)	125 (10.2%)		24 (5.9%)	144 (9.5%)		9 (8.5%)	159 (8.8%)	
Read & Write	77 (18.4%)	264 (17.6%)		139 (19.9%)	202 (16.6%)		89 (21.9%)	252 (16.7%)		19 (17.9%)	322 (17.8%)	
University	96 (22.9%)	362 (24.1%)		142 (20.3%)	316 (25.9%)		105 (25.8%)	353 (23.3%)		29 (27.4%)	429 (23.6%)	

The association between pain-related symptoms associated with Chronic Diseases and pilgrim’s characteristics. In this view back pain and foot pain were more common among women than men (p = 0.003), similarly foot pain was higher among pilgrims aged above 42 years (p = 0.001) as shown in Table 6.

**Table 6:** Association between Pain-Related Symptoms associated with Chronic Diseases and Pilgrim’s characteristics.

Variables	Abdominal pain		p-value	Back pain		p-value	Foot pain		p-value	Body ache		p-value
	Yes n(%)	No n(%)		Yes n(%)	No n(%)		Yes n(%)	No n(%)		Yes n(%)	No n(%)	
Gender												
Male	10 (50.0%)	938 (49.4%)	0.95	16 (29.6%)	932 (49.9%)	0.003	59 (37.1%)	889 (50.5%)	0.001	27 (45.8%)	921 (49.5%)	0.573
Female	10 (50.0%)	962 (50.6%)		38 (70.4%)	934 (50.1%)		100 (62.9%)	872 (49.5%)		32 (54.2%)	940 (50.5%)	
Age												
22–30	0 (0.0%)	109 (5.7%)	0.185	4 (7.4%)	105 (5.6%)	0.493	1 (0.6%)	108 (6.1%)	0.001	2 (3.4%)	107 (5.7%)	0.015
31–41	4 (20%)	342 (18%)		6 (11.1%)	340 (18.2%)		14 (8.8%)	332 (18.9%)		5 (8.5%)	341 (18.3%)	
42–52	3 (15%)	498 (26.2%)		14 (25.9%)	487 (26.1%)		42 (26.4%)	459 (26.1%)		10 (16.9%)	491 (26.4%)	
53–63	5 (25%)	570 (30%)		15 (27.8%)	560 (30.0%)		66 (41.5%)	509 (28.9%)		22 (37.3%)	553 (29.7%)	
>64 years	8 (40%)	381 (20.1%)		15 (27.8%)	374 (20.0%)		36 (22.6%)	353 (20.0%)		20 (33.9%)	369 (19.8%)	
Education level												
Missing	3 (15.0%)	805 (42.4%)	0.014	18 (33.3%)	790 (42.3%)	0.230	68 (42.8%)	740 (42.0%)	0.165	17 (28.8%)	791 (42.5%)	0.103
Illiterate	2 (10.0%)	143 (7.5%)		8 (14.8%)	137 (7.3%)		19 (11.9%)	126 (7.2%)		3 (5.1%)	142 (7.6%)	
Secondary	1 (5.0%)	167 (8.8%)		6 (11.1%)	162 (8.7%)		16 (10.1%)	152 (8.6%)		8 (13.6%)	160 (8.6%)	
Read & Write	9 (45.0%)	332 (17.5%)		8 (14.8%)	333 (17.8%)		24 (15.1%)	317 (18.0%)		16 (27.1%)	325 (17.5%)	
University	5 (25.0%)	453 (23.8%)		14 (25.9%)	444 (23.8%)		32 (20.1%)	426 (24.2%)		15 (25.4%)	443 (23.8%)	

## Discussion

It is worth noting that Hajj pilgrims face numerous challenges in completing the physically demanding rituals, in addition to the presence of various diseases, with a large proportion of pilgrims being elderly [20]. Other challenges included hot weather, congestion, and excessive physical exertion. Hence, it is imperative to assess the frequency of chronic illnesses and associated symptoms so that medical authorities and practitioners can create appropriate plans for managing or overcoming the illnesses and symptoms, ultimately contributing to a decrease in the number of fatalities from diseases in the sacred cities. By doing pre-health screenings before traveling to holy cities, these findings may additionally assist future pilgrims. In this study, the prevalence of Chronic Diseases in pilgrims performing Hajj was found to be 36.7%. However, the previous findings revealed that the presence of Chronic Diseases was more common among pilgrims [2,3,6,9]. For instance, a study by Mahmoud and Haroutune aimed to assess the epidemiology of Hajj pilgrimage mortality, the study revealed a 55.5% prevalence of Chronic Diseases [2]. Among South African pilgrims it was 48% [4], a further 45% of the pilgrims from Africa reported being ill during their stay in the Kingdom [4]. Similarly, in 2018, Hajj pilgrim’s statistics revealed that the prevalence of Chronic Diseases was 39.6%, with hypertension being the most reported comorbidity [10]. This difference in the prevalence of current and earlier studies can be explained by the fact that previous studies included patients admitted to the hospital, while the current study included only individuals who were performing the rituals at the holy cities.

The survey revealed that among the Chronic Diseases, hypertension accounted for 16%, diabetes for 11.6%, asthma for 4%, cardiovascular diseases for 3.4%, arthritis for 3.3%, and obesity for 0.7%. These results were in line with those of Yezli et al. from 2021, who found that between 1993 and 2018, the weighted pooled prevalence of hypertension was 12.2% among pilgrims and 5.0% among diabetics [9]. In a similar vein, Alrufaidi et al. found that 19.9% of the pilgrims had high blood pressure, 17.9% had asthma, 5.4% had myocardial infarctions, and 3.4% had cerebrovascular events [6]. In 2024, a study conducted on pilgrims participating in the Hajj revealed similar results, concluding that diabetes mellitus accounted for 31.6% of inpatient all-cause mortality, followed by hypertension at 24.8% and cardiovascular illnesses at 19.7% [2]. However, among French pilgrims, the prevalence of Chronic Diseases was reported to be markedly different, with 46.3% of the pilgrims being overweight, and 28.1% of them being obese, and the prevalence of diabetes mellitus and hypertension was 25.6% [21]. In addition, in the current study, the prevalence of liver, kidney, and lung diseases and obesity was less frequently reported among pilgrims [9,22]. These findings are in line with earlier findings where the authors reported that lung, renal, liver disease, cancer, and immune deficiency were very low. In addition, literature suggested a poor level of awareness, treatment, and control of various metabolic diseases and other underlying health conditions, especially in low- and middle-income countries, and a significant percentage of pilgrims with these conditions go untreated [9,22]. Therefore, it is advised to conduct sufficient health screenings for pilgrims, paying attention to how well the individual has controlled their chronic illnesses. Expanding these health screenings to undeveloped countries and villages all around the world, targeting pilgrims, would benefit them by enhancing their knowledge and awareness of the diseases and implementing adequate control measures [23].

In this study, 4.1% of the pilgrims reported to have vision impairment, while 0.8% of them revealed hearing impairment. In addition, mental health diseases like bipolar disorder, depression, and various forms of anxiety were reported by less than 1% among pilgrims. While a study by Khan SA, et al. revealed that 45.7% of the pilgrims had stress followed by 9.8% of their psychosis, 7.3% of insomnia, and 5.6% of them reported mood disorders [24]. Even though the majority of the pilgrims in this study were elderly, with a mean age of 51, it is typical for those over 50 to experience a variety of mental health issues in addition to visual and hearing impairment. Moreover, the majority of elderly individuals have at least one chronic disease, and a common symptom of many chronic diseases is eyesight impairment [18]. Furthermore, literature suggested that there is a strong correlation between depression symptoms and impairments related to hearing, vision, and dual sensory perception [18].

Even though the pilgrims in the current study had chronic illnesses, 36.5% of them reported having a cough, followed by fever (21.8%), sore throats (21.2%), and frequent sneezing (20.7%). However, a French study on pilgrims discovered that a significant percentage of pilgrims (93.4%) reported having at least one respiratory ailment, with cough and rhinitis being the most common, affecting 86.8% and 69.4% of pilgrims, respectively [21]. Furthermore, 27.3% of the pilgrims reported having a dry cough, and 37.2% reported having trouble speaking. 27.3% of the pilgrims said they had a fever [21]. Comparably, in a different survey conducted in 2022, out of 445 pilgrims, 4.7% experienced one or more respiratory conditions, with sore throat and cough being the most common. Moreover, the chance of getting RTIs was found to be significantly correlated with the presence of Chronic Diseases, including obesity [25].

Despite the Saudi Ministry of Health’s ongoing efforts to provide adequate healthcare to pilgrims, standard protocols for pre-healthcare screening and education about Chronic Diseases and their management before departure from home countries are required. In light of these findings, it is strongly recommended that countries educate and manage chronic disease patients before Hajj and local health organizations in Saudi Arabia, particularly in holy cities, maintain continuous and timely healthcare delivery, as well as awareness campaigns concerning symptoms associated with Chronic Diseases. These initiatives should concentrate on educating individuals and pilgrims about disease management through visits to healthcare facilities in holy cities. By raising awareness and communicating critical information, providing healthcare to pilgrims can help to reduce the frequency of Chronic Disease-related mortality and morbidity during the Hajj.

Nevertheless, there are various limitations to this study. Initially, the study population was drawn at random from the holy cities’ designated areas; it did not include all pilgrims. As such, it might not accurately represent the long-term medical conditions of every pilgrim who performed the Hajj in 2024. Second, the sample size may be regarded as small because the pilgrims in our study had a very low response rate. Third, the study’s responses might not accurately represent the illnesses’ actual symptoms because it used an online survey approach administered by an interviewer. However, since the study was conducted during the Hajj when pilgrims were already finding it difficult to perform the rituals both physically and mentally, this could be one of the reasons why there are so many symptoms connected to persistent diseases. Future studies examining the effects of chronic illnesses and their symptoms on health outcomes and mortality during Saudi Arabia’s Hajj seasons are required.

## Conclusion

A considerable number of pilgrims suffer from Chronic Diseases, with hypertension being the most common. Further interventions, such as education and management before Hajj, are recommended. With current interventions to address the increased risk of pilgrims with pre-existing diseases, further steps need to be taken to assess pilgrims and assist with the management of clinical symptoms to allow pilgrims to complete Hajj.

## Supporting information

S1 Data Data collected to explore the prevalence of chronic diseases and health status among International Hajj pilgrims. (XLSX)
